# Tunneling Nanotubes and the Eye: Intercellular Communication and Implications for Ocular Health and Disease

**DOI:** 10.1155/2020/7246785

**Published:** 2020-04-08

**Authors:** Holly R. Chinnery, Kate E. Keller

**Affiliations:** ^1^Department of Optometry and Vision Sciences, The University of Melbourne, Monash Road, Parkville 3010, Australia; ^2^Department of Ophthalmology, Casey Eye Institute, Oregon Health & Science University, 3181 SW Sam Jackson Park Road, Portland, OR, USA 97239

## Abstract

Cellular communication is an essential process for the development and maintenance of all tissues including the eye. Recently, a new method of cellular communication has been described, which relies on formation of tubules, called tunneling nanotubes (TNTs). These structures connect the cytoplasm of adjacent cells and allow the direct transport of cellular cargo between cells without the need for secretion into the extracellular milieu. TNTs may be an important mechanism for signaling between cells that reside long distances from each other or for cells in aqueous environments, where diffusion-based signaling is challenging. Given the wide range of cargoes transported, such as lysosomes, endosomes, mitochondria, viruses, and miRNAs, TNTs may play a role in normal homeostatic processes in the eye as well as function in ocular disease. This review will describe TNT cellular communication in ocular cell cultures and the mammalian eye *in vivo*, the role of TNTs in mitochondrial transport with an emphasis on mitochondrial eye diseases, and molecules involved in TNT biogenesis and their function in eyes, and finally, we will describe TNT formation in inflammation, cancer, and stem cells, focusing on pathological processes of particular interest to vision scientists.

## 1. Introduction

**Figure 1:**
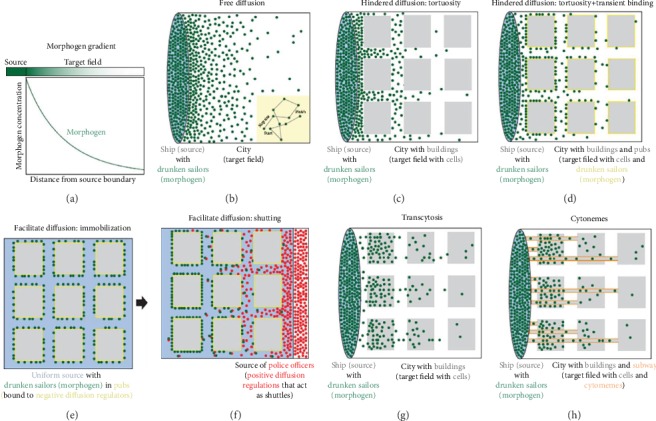
Morphogen transport and the drunken sailor analogy. (a) The transport of morphogens from a source establishes a gradient in the target field. (b–h) Five major morphogen transport models are illustrated using the drunken sailor analogy, in which drunken sailors move by random walks from a ship into a city. In this analogy, morphogen molecules are represented by sailors and cells are represented by buildings. (b) In the case of free diffusion, sailors (green dots) leave the ship (blue oval) and disperse into the city (white square). Inset: sailors take steps of the indicated fixed size, and the direction of each step is random. This “random walk” describes the diffusive behavior of molecules in solution. (c) In the tortuosity-mediated hindered diffusion model, buildings (gray) act as obstacles that sailors must move around, thus increasing the tortuosity of the environment. (d) In the case of diffusion that is hindered by tortuosity and transient binding, the sailors stop in pubs (negative diffusion regulators, yellow) located at the periphery of buildings. Note that, in contrast to effects from tortuosity alone, sailors congregate at the periphery of buildings, and there are relatively few freely moving sailors. (e, f) The shuttling model does not require a localized source of sailors. Instead, sailors are initially present mostly in pubs (negative diffusion regulators, yellow) and uniformly distributed in the city (e). Police officers (positive diffusion regulators, red) disperse from a source on the right side, pick up sailors from pubs, and escort them through the city by preventing further pub visits (f). When police officers disappear (not shown), sailors can reenter the pubs. Over time, this results in the concentration of sailors on the left. (g) In the transcytosis model, the sailors travel through the buildings. (h) During directed transport-mediated by cytonemes, the sailors travel through subway tunnels (orange), which deposit the sailors in buildings (reproduced from Muller et al. (2013) with no alterations under the Creative Commons Attribution 4.0 International license (http://creativecommons.org/licenses/by/4.0/)) [1].

**Figure 2:**
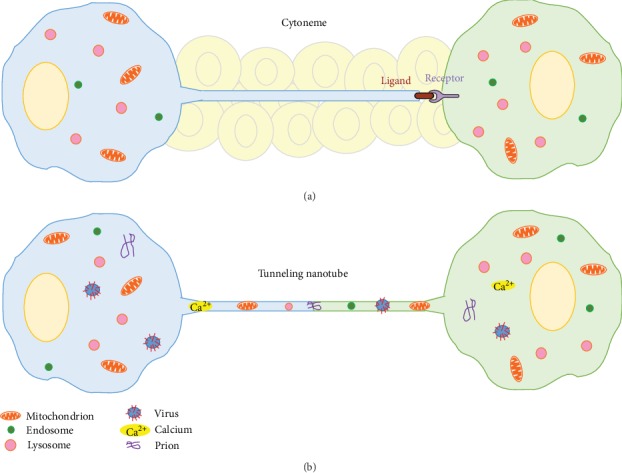
Cytonemes and tunneling nanotubes. (a) Cytonemes were originally identified in Drosophila imaginal discs but have also been detected in developing chick limb buds. These cellular structures transport ligands and receptors over long distances from one cell to another in the complex tissue environment. Ligands cluster at the tip of a cytoneme and interact with clustered receptors on the surface of the recipient cell. (b) Tunneling nanotubes (TNTs) transport cellular organelles such as mitochondria, endosomes, and lysosomes, as well as other cargoes such as viruses, prions, and Ca^2+^ signals. TNTs form a tube through which these cargoes are directly transported between cells.

Cells have numerous mechanisms of communication in order to develop normally, repair wounds, and respond to inflammation. Many of these mechanisms rely on secretion of signals from one cell, diffusion through the extracellular milieu, and uptake by a target cell, which may be located far from the secretory cell. An excellent synopsis of morphogen signaling was recently published, which uses a “drunken sailor” analogy to comprehensively describe the current models, hypotheses, and challenges of diffusion-based signaling processes (Figure 1) [1]. Two more recent modes of communication are also described: cytonemes and tunneling nanotubes (TNTs) (Figure 2). These cellular structures allow signaling over long distances by providing direct connections between cells, which overcomes many challenges of diffusion-based signaling [2–7]. While cytonemes have been described in the *Drosophila* imaginal eye disc [8], nothing is known of their existence in the vertebrate eyes. Conversely, there is a burgeoning literature on TNTs since their first description in 2004 [2]. TNTs are specialized filopodia that transport signals directly between cells. TNTs are composed of an actin core, although microtubules may also be involved in certain cell types [9]. Due to continuity of cytoplasm within the tube, larger cargo can be trafficked than could be shared via gap junctions [10–14]. LysoTracker-labeled vesicles were the first organelles shown to be transported from one cell to an adjacent one [2]. Other groups subsequently showed the intercellular transfer of mitochondria and endocytic vesicles derived from early endosomes, the Golgi complex, endoplasmic reticulum, and lysosomes [15–20]. In addition, TNTs are involved in the spread of pathogens such as viruses, prions, and bacteria [12, 18, 21–23] and other small molecules such as miRNAs, Ca^2+^, calcein, Lucifer yellow, and quantum dot nanoparticles [11, 24–29]. However, there appears to be some selectivity in the cargo that is transferred because some TNTs support transfer of electrical signals, while other cell types do not [10, 30]. Thus, TNTs may be responsible for communicating cellular signals that previously were thought to have been mediated by diffusion.

There are several excellent reviews that provide in-depth discussion on TNT biogenesis [11, 19, 30, 31]. Two current biogenesis models are the cell dislodgement and the actin-driven models [2, 3, 25]. In the former, two adjacent cells in close proximity form short tubes, which are drawn out as the cells move apart. In the actin-driven model, filopodia extend from two adjacent cells and when their tips touch, they fuse to form a single conduit. As the name implies, this latter mechanism depends on actin. Several molecules are also implicated in TNT formation, including M-Sec, myosin-Va, myosin-X, synaptophysin, Rho GTPases (cdc42, Rac1), RASSF1A, LST1, and Rab8/Rab11a [25, 32–39].

This review will detail the current knowledge of the existence and potential role of TNTs in ocular homeostasis and disease pathogenesis. We will first describe (1) the current knowledge of TNTs in the mammalian eye, (2) the role of TNTs in mitochondrial transfer with an emphasis on eye disease, (3) TNT molecular regulators and their function in eyes, and (4) the role of TNTs in other diseases that are of interest to vision researchers.

## 2. TNTs and Cytonemes in the Eye

### 2.1. TNTs in Corneal Tissue

**Figure 3:**
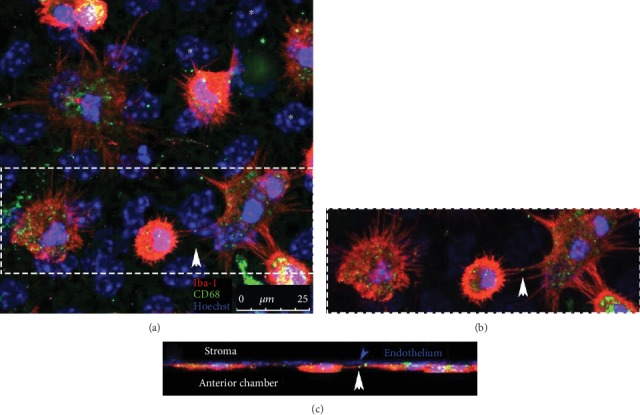
TNTs connecting inflammatory macrophages adhering to the corneal endothelium. (a) Confocal Z-stack of Iba-1^+^ (red) CD68^+^ (green) macrophages on the posterior surface of the corneal endothelium in mice one week after topical exposure to synthetic DNA, which causes accumulation of keratic precipitates [136] (asterisks denote nuclei of the endothelial cell monolayer). Interconnecting cytoplasmic TNTs are clearly visible between some of the macrophages (b, inset of dashed box in a), with punctate intracellular CD68^+^ signal visible between two cells (arrows; which represent approximately 3 *μ*m distance from the endothelium). (c) Rotated view of Z-stack showing nanotube connecting the two cells (white arrow in c), which appears above the layer of the endothelium (blue arrow in c). Scale bar is 25 *μ*m.

A few years following the first reports of TNTs in *in vitro* settings, Chinnery et al. described the presence of long (up to 300 microns), thin (<1 micron) membrane extensions protruding from MHC Class II-positive immune cells in the mouse cornea (Figure 3) [40]. The transparent tissue environment of the corneal stroma, with its unique arrangement of collagenous lamellae and absence of pigmentation, likely enabled the first visualization of these elusive cellular structures in a mammalian tissue. Unlike other tissues such as the skin, where resident tissue macrophages and dendritic cells are abundant (approximately 1000 cells/mm^2^) [41], the density of immune cells in the cornea is comparatively low (100-150 cells/mm^2^) [42]. Thus, intercellular communication between widely spaced cells in the cornea may be facilitated by the presence of long cellular extensions. In a follow-up study, membrane nanotubes were also identified in the mouse dura mater, which is another dense connective tissue that shares similar structural features with the cornea [43]. Using time-lapse confocal microscopy, nanotube formation in the mouse cornea was dynamically visualized in corneal explants from macrophage GFP reporter mice, with evidence supporting *de novo* formation, with an average speed of 15 *μ*m/minute.

While the first two reports of TNTs in the mammalian cornea were novel, insights into their proposed function or role in disease were not provided. In a study exploring the potential for treating corneal pathology associated with the lysosomal storage disease, cystinosis, Rocca and colleagues performed a hematopoietic progenitor stem cell transfer from healthy wild-type donor mice into cystinosin gene-deficient animals (ctns-/-) [44]. In addition to improving the clinical outcome of ocular pathology, such as corneal thinning and elevated IOP, transfer of transplanted cystinosin lysosomes from healthy donor cells into diseased, recipient cells was observed in corneal macrophages. Macrophage-mediated transfer of healthy cystinosin lysosomes to diseased fibroblasts via TNTs was also demonstrated *in vitro* [45]. These studies provide compelling evidence that damaged intracellular organelles can be “rescued” by transplantation of healthy donor cells, with TNTs serving as conduits for delivery of the healthy cargo.

### 2.2. TNTs in the Trabecular Meshwork

The trabecular meshwork (TM) is a small, circumferential, sieve-like tissue located in the anterior segment of the eye. The TM regulates aqueous humor outflow from the anterior chamber and thus establishes intraocular pressure (IOP) [46]. When outflow is disrupted, due to blockages in the TM outflow channels, IOP begins to increase. Elevated IOP is a primary risk factor for glaucoma, a leading cause of blindness worldwide [47]. TM cells sense changes in IOP and communicate signals to remodel the extracellular matrix to allow greater aqueous fluid flow and alleviate pressure. However, signaling in this aqueous environment is challenging because signals secreted from the cell are immediately diluted in aqueous humor fluid, which can drain into Schlemm's canal before reaching their target cell. Furthermore, cells in the putative stem cell region of the tissue, posterior to Schwalbe's line, are not bathed in aqueous humor [48]. Yet, we know that these cells must receive signals because they are induced to migrate into the TM in response to burns placed by laser trabeculoplasty, a common treatment to relieve elevated IOP in glaucoma patients [49]. It is currently unclear how signals released at laser sites can be transported long distances (>100 *μ*m) at high enough concentrations to elicit their effects at the insert region. This suggests additional mechanisms must be employed by TM cells to communicate signals in an aqueous environment.

**Figure 4:**
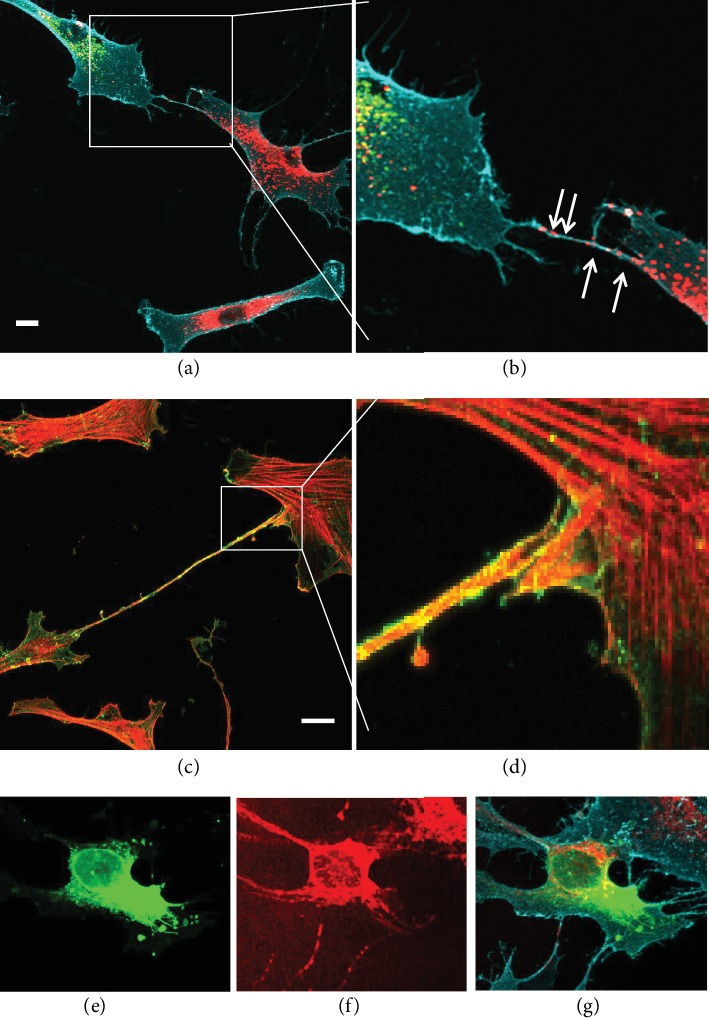
Cellular protrusions formed by ocular trabecular meshwork (TM) cells. (a, b) The cell membrane of TM cells was labeled with CD44 antibodies (cyan), and vesicles were labeled with DiO (green) or DiD (red). DiD-labeled vesicles are clearly observed in a TNT connecting to a DiO-labeled cell. (c, d) A long cellular protrusion (130 *μ*m) extends between two TM cells in culture (CD44 immunostaining = green; SiR-actin = red). At higher magnification, the tips of the protrusion are not fused to form a continuous tunnel so this structure may be a cytoneme. (e–g) TM cells were labeled with DiO (e; green) or with MitoTracker (f; red) and CD44 cell membrane immunostaining (cyan). Red mitochondria are transferred into a DiO-labeled cell via TNTs. Scale = 20 *μ*m.

TNTs formed by cultured TM cells were first described by Keller et al., in 2017 (Figure 4) [50]. In this study, live cell imaging showed the transfer of DiO fluorescently labeled vesicles and mitochondria from one TM cell to another. Over the course of 40 minutes, four vesicles were transferred. Using various manipulations of the actin cytoskeleton, a Rho kinase inhibitor, Y27632, increased the number of vesicles transferred, while inhibition of the Arp2/3 complex with CK-666 reduced vesicle transfer [50]. When glaucoma TM cells were compared to normal TM cells, TNTs were less abundant, but they were longer [51]. Also, the actin cytoskeleton was less dynamic in GTM cells and it appeared that transfer may be bidirectional since both DiO- and DiD-labeled vesicles were present in the same TNT. A greater number of GTM cells contained vesicles of the opposite color compared to normal TM cells, suggesting that a more stabilized actin cytoskeleton could lead to prolonged TNT connections, which, combined with possible bidirectional vesicle transfer, would account for the increased transfer over time. Together, these data provided the first evidence that normal and glaucomatous TM cells utilize TNTs to communicate with each other.

### 2.3. TNTs in the Retina

The retinal pigment epithelium (RPE) is a monolayer of pigmented cells that provides metabolic support to the photoreceptors in the retina. TNTs have been detected in cultured RPE cells [52]. In these cells, TNTs transferred mitochondria, endosomes, and Ca^2+^. Transfer of Ca^2+^ indicates that the TNTs were electrically coupled, which was confirmed by the localization of connexin 43+ gap junctions at the tips of TNTs connecting RPE cells [52]. However, gap junctions cannot transfer larger molecules so another mechanism must exist for organelle transfer. As well as RPE cell communication, TNTs may also play a role in angiogenesis in the retina. A recent study showed that vascular endothelial growth factor (VEGF) and hypoxia-inducible factor-1*α* (HIF-1*α*) signals can be transferred between malignant ovarian cancer cells and human vascular endothelial cells (HUVEC) [53]. The authors speculated that TNTs could stimulate angiogenesis by propagating these potent angiogenic factors. If similar studies showed that VEGF and HIF-1*α* were transported by TNTs in retinal cells, a new target for anti-VEGF treatments for diabetic retinopathy, retinopathy of prematurity, and/or wet age-related macular degeneration may be revealed.

Pericytes are mesenchymal-derived cells that encircle endothelial cells of capillaries in the microvasculature. Together with astrocytes and neurons, they form a neurovascular unit that functions to maintain the blood-brain and blood-retina barrier [54]. Retinal pericytes are lost in diabetic retinopathy patients, making retinal capillaries vulnerable to proliferation signals and formation of leaky neovessels which can lead to blindness [55]. In the brain, pericytes form TNTs, which appear to function in probing the environment for other cells as well as communicating signals during brain development and during pathological neovascularization [56]. The role of pericyte-derived TNTs in the retina is an unexplored target to prevent abnormal angiogenesis, which underpins the pathogenesis of several blinding diseases affecting the retina.

### 2.4. Cytonemes in the *Drosophila* Eye

Like TNTs, cytonemes are specialized filopodia with an actin core (Figure 2). However, instead of forming a tube through which cargo is carried, signals travel along the surface of the cytoneme [7]. Cytonemes were first described by Ramirez-Weber and Kornberg in 1999, who studied decapentaplegic signaling in the *Drosophila* wing imaginal disc [6]. Later studies investigated epidermal growth factor (EGF) signaling in the *Drosophila* eye disc and found that epithelial cytonemes, which express the EGF receptor, orient themselves toward the morphogenetic furrow, where the Spi/EGF-producing cells are located [8]. Thus, cytonemes extend long distances (up to 20 cell diameters) to link EGF producing and receiving cells, which provides specificity in signaling over long distances in tissues [57]. To date, there are no descriptions of cytonemes in the vertebrate eye. Recent reviews describe the similarities and differences between cytonemes and TNTs in more detail [58, 59].

## 3. Mitochondrial Intercellular Transfer via TNTs and Implications for Eye Diseases

Mitochondrial dysfunction in ocular disease is relatively common, especially in genetic disorders that disrupt their structure or function [60]. Mutations in *OPA1* and *MFN2* genes cause autosomal dominant optic atrophy (DOA); mutations in *WFS1* and *CISD2* can cause Wolfram syndrome, while Leber's hereditary optic neuropathy (LHON) is associated with mutations in mitochondrial *ND1*, *ND4*, and *ND6* genes [61, 62]. However, many other eye diseases have been linked to mitochondrial dysfunction occurring secondary to other conditions including glaucoma and other optic neuropathies, age-related macular degeneration, diabetic retinopathy, and retinitis pigmentosa [63–71].

Intercellular transfer of mitochondria can occur via TNTs, and it appears to be a common occurrence in cell cultures and possibly *in vivo* [72, 73]. Mitochondria are ATP generators, and they are essential for cell survival. The transfer of mitochondria between cells was first observed in coculture experiments where healthy mitochondria from human mesenchymal stem cells were transferred via cytoplasmic projections to A549 cells, which have defective or depleted mtDNA [74]. Several subsequent studies suggested that intercellular transfer of mitochondria is linked to cell survival [16, 17, 75–77]. For instance, Liu et al. [16] showed that mesenchymal stem cell-derived mitochondria rescued oxidatively stressed human umbilical vein endothelial cells (HUVECs) from apoptosis. Other groups have suggested that TNT-mediated transfer could induce differentiation of mesenchymal stem cells into renal tubule cells [27], while mitochondrial transfer in cocultures of endothelial cells and cancer cells conveyed chemoresistance to the cancer cells receiving the mitochondria [78]. Transcellular mitochondrial exchange is not limited to cells in culture with new evidence showing that this process can also occur *in vivo* where healthy mitochondria were transferred from astrocytes to neurons in the mouse brain [76]. This transfer was CD38-dependent and led to a neuroprotective effect after stroke. While these *in vitro* and *in vivo* studies show mitochondrial transfer between heterotypic cell types, other groups describe mitochondrial transfer between homotypic cell types [20, 50, 79]. For instance, Wang and Gerdes demonstrated that mitochondria transferred from untreated, healthy PC12 to UV-stressed PC12 cells reversed the early stages of apoptosis [20]. Little is known about the molecules that are involved in mitochondrial trafficking, but Miro1 appears to be involved [80].

A role for mitochondrial transfer in ocular cells is emerging. In the cornea, mitochondria were transferred via TNTs in cocultures of corneal epithelial cells and mesenchymal stem cells [81]. When MSCs were applied to the corneas of rabbits with alkali burns, mitochondria were transferred to the host corneas. However, it could not be determined whether this transfer was via TNTs or whether it was due to release of mitochondria into the extracellular space for uptake by neighboring cells. In retinal ARPE-19 cells, mitochondria and endocytic organelles were found within the TNT connections [52]. With this discovery, the authors suggested that mitochondria may be transferred between themselves or hypothetically between RPE cells and photoreceptors [52]. While RPE cell culture experiments show TNTs, TNTs in retina tissue *in vivo* have not yet been reported. This is likely due to their fragility upon fixation and lack of a good biomarker. However, one study hints at their existence. Transcellular mitochondrial exchange between neurons and glia in the optic nerve head (ONH) was recently described [82]. In this study, serial block-face scanning electron microscopy demonstrated that mitochondria shed from ONH-resident neurons were engulfed and degraded by lysosomes in neighboring astrocytes. It is possible that direct connections via TNTs were involved in this mitochondrial transfer, but the thin tubules may have been destroyed during the harsh fixation treatments used in the preparation procedures used for electron microscopy. While speculative, studies such as these are highlighting the importance of intercellular transfer of mitochondria and TNTs as a potential mechanism of transfer.

## 4. Molecular Regulation of TNTs in the Eye

Several molecules play a role in TNT biogenesis, and some of these have roles in various physiological processes in the eye. For instance, Rab8a/Rab11a and myosin-Va, which are associated with TNTs [2, 37], have been found to be involved in normal photoreceptor signaling in the retina [83, 84]. In *Xenopus* and zebrafish photoreceptors, Rab8a/Rab11a are involved in transporting rhodopsin from the Golgi to the distal inner segment membranes, the first step before intraflagellar transport to the outer segment. However, the role of Rab8a/Rab11a in the mammalian eye appears more complex since they are dispensable for the transport of rhodopsin to the mouse outer segment [84]. Further studies are needed to resolve the contribution of these proteins to TNT biogenesis and normal homeostatic processes in the eye. However, one TNT regulator, myosin-X (Myo10), appears to play a major role in TNT-mediated processes in the eye, which we will now describe in more detail.

### 4.1. Myo10 Background

Several groups have described a role of Myo10 in filopodia and TNT formation [85–87]. Myo10 belongs to a group of four unconventional myosins—Myo7a, Myo7b, Myo10, and Myo15a—which, unlike conventional myosins, do not form filaments and are not involved in muscle contraction [88]. Myo10 was first described by Berg et al. [89] and is localized to areas of dynamic actin reorganization. It is composed of an actin-binding head domain, a neck domain, and a tail domain, which is involved in binding of cargo and dimerization [90]. The head domain of Myo10 binds to F-actin and acts as a molecular motor, hydrolyzing ATP to transport itself along the actin filaments. The head domain is essential for this transport since “headless” recombinant constructs do not travel along filopodia [91]. Some cell types such as neurons express an endogenous “headless” Myo10, which negatively regulates full-length Myo10 [92, 93]. The neck domain of Myo10, which contains IQ motifs, may regulate the motor activity of the head domain or increase flexibility of the molecule as it walks hand-over-hand along actin filaments [94]. The tail domain offers diversity from the other unconventional myosins and is composed of a coiled-coil region, three PEST domains, three PH domains, a myosin tail homology 4 (MyTH4), and a band 4.1, ezrin, radixin, moesin (FERM) domain [90]. The coiled-coil domain is important for dimer formation. The antiparallel coiled-coil structure of Myo10 dimers is optimized to move on actin bundles rather than single actin filaments [95]. The MyTH4 and FERM domains in the tail domain also bind several different molecules including microtubulin, *β*-integrin, and the VE-cadherin complex [96–98].

### 4.2. Myo10 Role in Filopodia and TNT Formation

Overexpression of Myo10 in a variety of cell types increases the number of filopodia emanating from the cell surface, while Myo10 knockdown reduces filopodia number [85, 86, 91, 99, 100]. Overexpression of Myo10, but not VASP or fascin, also induced TNT formation [87]. Other studies show that Myo10 appears to play multiple roles in filopodia/TNT formation including at the initiation and elongation phases [86, 101]. Shorter filopodia predominated when the C-terminal FERM domain was eliminated [86]. Live cell imaging using GFP-tagged Myo10 constructs shows Myo10 in bright puncta at the filopodia tips and along the length of the cellular protrusion [90, 91]. Fainter Myo10 clusters were also apparent along the filopodial shaft. The bright puncta travel at around 80 nm/second, while the faint Myo10 moves even faster at around 600 nm/sec [90]. Myo10 also undergoes retrograde movement but at much slower speeds of 15 nm/sec, a rate that is similar to the retrograde flow of actin [102]. There is some controversy of whether Myo10 moves faster on actin filaments or bundles [95, 103, 104]. Differences may be due to the origin of actin bundles used in each study, i.e., fascin-induced actin bundles versus nascent filopodia, which presumably contain additional actin-binding proteins. Together, these studies demonstrate that Myo10 plays a critical role in TNT biogenesis, elongation, and cargo transport.

### 4.3. Eye Phenotypes Caused by Myo10 Knockdown and Knockout

Myo10 is expressed in the retina and the TM [105–107]. Recent RNAi knockdown studies and knockout mouse models have revealed functional roles for Myo10 in the eye. In an anterior eye study, RNAi silencing lentivirus was generated to selectively knock down Myo10 in TM tissue and cells [107]. First, Myo10 silencing lentivirus was applied to an *ex vivo* organ culture perfusion model to examine the effects on outflow and IOP regulation [107]. Outflow rates were significantly reduced suggesting that Myo10-mediated filopodia/TNTs play a role in IOP regulation. In Myo10-silenced TM cell cultures, matrix metalloproteinase activity was reduced, consistent with a reduction in focal remodeling of the extracellular matrix components of the outflow resistance. Of note, Myo10 protein distribution in glaucomatous TM tissue was disrupted compared to age-matched normal tissue [51].

**Figure 5:**
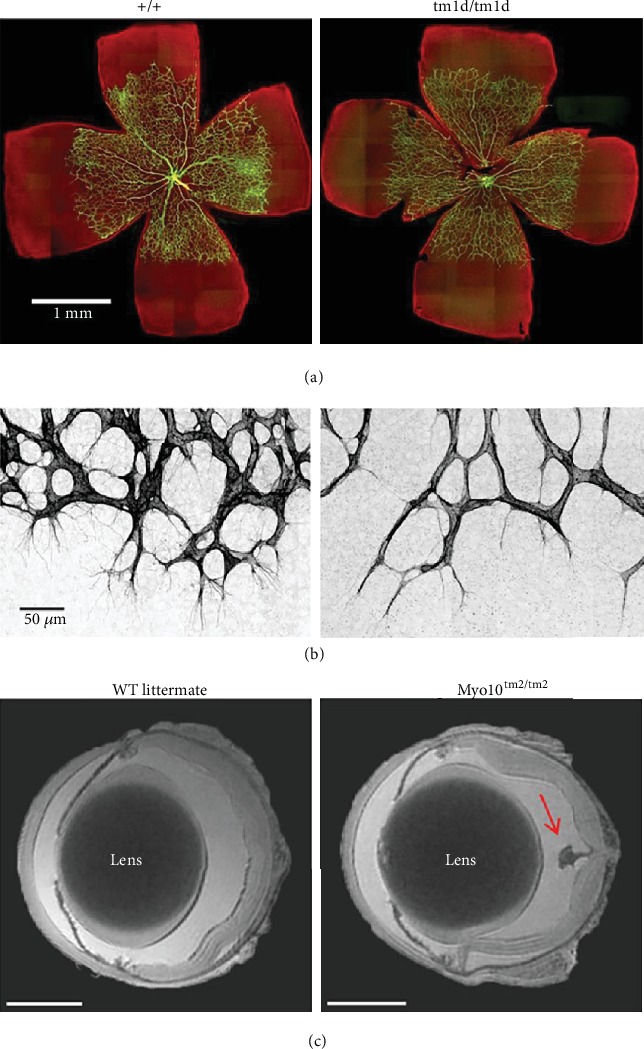
Phenotype of two *Myo10* knockout mouse models. *tm1d* completely ablated all Myo10 forms, whereas *tm2* selectively knocked out the full-length, actin-binding Myo10, but “headless” Myo10 expression was unaffected. (a) Fluorescence image of flat mounted retinas showing retinal vasculature at P5. Dissected retinas were stained with a PECAM-1 antibody (green) and counterstained with phalloidin (red). The retinal vasculature extended to similar positions in the control and *Myo10^tm1d/tm1d^* eyes. The image represents a stitch of micrographs taken at 20x and is best visualized if the images are enlarged on a digital display. (b) High-resolution images of the angiogenic expansion front from the P5 retinas in (a) showing filopodia radiating from endothelial tips cells. Loss of *Myo10* results in a decreased number of filopodia and leads to a less dense vascular network. Images were captured as Z-stacks at 60x and displayed as maximum projections with the PECAM-1 channel displayed in inverted grayscale to highlight endothelial filopodia. (c) High-resolution MRI (magnetic resonance imaging) of enucleated and fixed eyes from adult wild-type (WT) and *Myo10*^tm2/tm2^ mice, where representative of 6 eye scans for each genotype reveals persistence of the hyaloid vasculature in mutant mice. The hyaloid artery emerges from the optic disc and extends toward the lens, as schematically illustrated on the right. Scale bars: 1 mm (reproduced from (a, b) Heimsath et al. 2017 and (c) Bachg et al. 2019 under the Creative Commons Attribution 4.0 International license (http://creativecommons.org/licenses/by/4.0/)) [105, 106].

Two Myo10 knockout mouse models were recently described, and both have posterior chamber ocular phenotypes (Figure 5) [105, 106]. The first knockout model ablated both full-length and headless forms of Myo10 [105]. Between embryonic days E12.5 and E17.5, approximately 60% of Myo10 knockout mice exhibited exencephaly, a lethal neural tube closure defect. However, 40% of homozygotes survived to adulthood. Of these survivors, all exhibited a white belly spot and persistent fetal vasculature (PVF) in the eye (Figures 5(a) and 5(b)). Other less penetrant phenotypes included kinked tails and soft tissue syndactyly of the digits. The bilateral persistent hyaloid vasculature in the posterior eye was phenotypically similar to the human disease PVF. In normal mice, the hyaloid vasculature develops around E12.5 and is resorbed soon after birth (2-3 weeks). In Myo10 knockout mice, hyaloid regression was dysfunctional and a thin strand, which emanated from the optic disc, extended through the vitreous, and connected to the posterior lens, persisted. Retinal angiogenesis was also delayed in Myo10-/- mice. Analysis of the retina using the endothelial biomarker, PECAM-1, showed that the retinal vascular network was less dense, and the endothelial cells had 50% less filopodia (Figure 5) [105]. Other eye defects such as embryonic microphthalmia, anophthalmia, an optic fissure closure defect, cloudy cornea, and lens spots were noted, but these were sporadic [105].

A second Myo10 knockout mouse was developed, which selectively knocked out the full-length Myo10 molecule, the actin-binding form, but “headless” Myo10 expression was unaffected [106]. Similar to the complete Myo10 knockout, persistent hyaloid vasculature, white belly spots, and syndactyly were common, but there was a reduced rate of exencephaly (24%) (Figure 5(c)). Surprisingly, there was no vascularization defects noted despite Myo10 being the most strongly expressed unconventional myosin in retinal vascular endothelial cells. This selective knockout shows that full-length Myo10 is important for hyaloid regression, but not retinal vascularization. Together, these mouse models indicate that Myo10 is required for normal embryonic development and is important for filopodia formation *in vivo*.

## 5. Role of TNTs in Diseases Relevant to Vision Research

In addition to their potential role in ocular disease, TNTs are involved in many other pathological disease processes. Several recent review papers provide discussion of TNTs in health and disease in greater detail [39, 108–111]. Here, we review the role of TNTs in several pathological processes of particular interest to vision scientists.

### 5.1. Inflammatory Responses

Acute and chronic inflammation underpins a wide variety of ocular diseases, which can have both local and systemic origins. Inflammation is a known stimulator of TNT formation in immune cells [40, 112], but the precise functional significance of this is unclear. Theoretically, TNT could be exploited *in vivo* to deliver immunomodulating proteins that could inhibit or promote inflammation, depending on the therapeutic need. In a study on the feasibility of synthetic nanotubes as a drug delivery system, peptide nanotubes were designed to deliver Caspase-3 siRNA as a means to inhibit keratocyte apoptosis in the injured mouse corneal stroma [113]. While this study was based on exogenous nanotube delivery to the wounded cornea, it does provide evidence that nanotube-mediated transfer of therapeutic siRNA could occur *in vivo*. *In vitro* evidence suggests that when fibroblasts are cocultured with TGF-*β*1-stimulated dendritic cells, membrane nanotube formation increases. Furthermore, tenascin-C, a proregenerative extracellular matrix molecule, was localized to the TNTs [114], suggesting that exposure to certain growth factors can promote the formation of TNTs between fibroblasts and possibly contribute to tissue remodeling. Whether the delivery of drugs or therapies aimed at suppressing ocular inflammation could also apply to biological TNTs, either through donor cell transfer or by upregulating endogenous TNT formation *in vivo*, is a largely unexplored and exciting future area of research.

### 5.2. Cancer

Several studies have investigated the role of TNTs in cancer (reviewed in [111]). Of particular interest to vision researchers is the role of the tumor suppressor gene, *RASSF1A*, in uveal melanomas [115]. *RASSF1A* binds cytoskeletal proteins such as actin and microtubules, playing an important role in TNT formation, cell cycle regulation, and apoptosis to maintain cellular homeostasis [35, 111]. Loss of *RASSF1A* gene expression is observed in certain cancers, including uveal melanomas in the eye [115, 116]. In malignant mesothelial cell lines, downregulation of *RASSF1A* increased the number of TNT connections, while overexpression decreased the TNT number and length [35]. Thus, increased TNT-mediated communication concomitant with downregulation of *RASSF1A* appears linked to cancer progression. *RASSF1A* is downregulated in uveal melanoma tumors as well as in uveal melanoma cell lines [115]. Therefore, it seems plausible that TNT communication is involved in uveal melanomas.

### 5.3. Stem Cells

Therapeutic stem cell transplantation is a hot area of vision research. Recent evidence suggests that stem cells may not necessarily integrate as functioning cells but rather exert a local trophic influence to regenerate host cells [117]. *In vitro* studies have reported the transfer of mitochondria and other cellular organelles between heterotypic cell types such as mesenchymal stem cells and endothelial cells [16, 17, 45, 75, 81, 118–120]. Many of these studies also describe that formation of TNT connections “rescues” dying cells. In the cornea, hematopoietic stem progenitor cells (HSPCs) were transplanted into cystinosin-null mice and these were found to home to the cornea to restore normal corneal structure and function [44]. Interestingly, some of these HSPCs differentiated into macrophages, which formed TNTs that appeared to transport cystinosin-containing lysosomes into adjacent diseased cells. Thus, control of TNTs may provide a novel mechanism to transport normal proteins to diseased cells in lysosomal storage diseases [121].

Transplantation of stem cells has also been suggested as a therapeutic option for glaucoma [48, 122]. Stem cells may provide a source of factors that promote survival of resident cells or modulate the intraocular microenvironment. Mesenchymal stem cell (MSC) transplantation has shown to be neuroprotective for RGCs [122], and MSCs offer an attractive therapeutic option for repopulating the cells of the TM of POAG patients, which have a marked reduction in the TM cell number [123]. Several groups have shown that introduction of MSCs [124], or induced pluripotent stem cells (iPSCs) [125–127], may restore TM IOP regulation function. In both anterior and posterior glaucoma stem cell therapies, it is conceivable that TNTs formed between stem cells and resident cells could promote survival of the remaining endogenous host cells. Further studies are required to explore these possibilities.

## 6. Future Perspectives: Potential Applications of TNTs in Ocular Therapies

A growing area of ocular research concerns the delivery of drugs and gene therapies [128–131]. Many papers have described the use of nanoparticles as drug delivery systems, which offer attractive advantages over conventional methods including targeted delivery, increased drug bioavailability with lower doses, sustained release, and reduced side effects [132]. Nanoparticles can be used to deliver a variety of payloads such as ocular gene therapies, contrasts agents for bioimaging, nanodrugs, and/or antimicrobial agents [133]. He et al. reported that quantum dots made from CdSe/ZnS (15-20 nm diameter) were rapidly internalized by cardiac myocytes and were transported bidirectionally via TNTs [24]. Subsequently, other researchers showed that nanoparticles made from porous silicon (3.2 *μ*m diameter) were directly transferred between cells via TNTs [134]. These studies in other tissues of the body suggest that TNTs may be used to deliver nanoparticles to specific tissues in the eye.

Viruses can spread via TNTs [7], which could be exploited to deliver viral-mediated gene therapies in the eye. Recently, the first gene therapy, Luxturna, received FDA approval. This modified, inactive AAV2 virus is delivered by subretinal injection to replace defective RPE65 in children with Leber's congenital amaurosis [135]. Since RPE cells *in vitro* communicate via TNTs [52], it is conceivable that TNTs could be utilized to provide more effective delivery of virus-mediated gene therapies to targeted photoreceptor cells in the retina. However, multiple challenges remain and more research is needed to reveal how TNTs can be manipulated to form *de novo* to ensure efficient transport of the nanoparticles or viruses *in vivo*.

## 7. Final Thoughts

**Figure 6:**
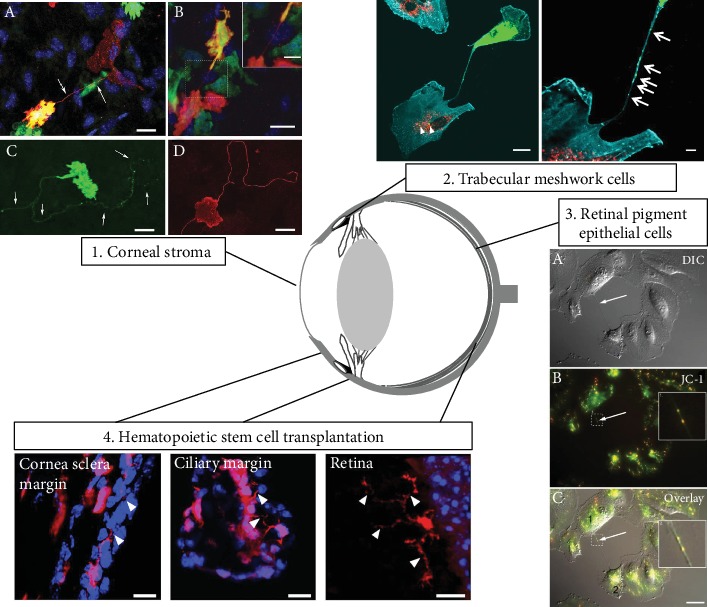
Schematic of the eye summarizing the current findings on TNTs in various anterior and posterior ocular cells and tissues. TNTs are found in (1) mouse corneal stroma [40], (2) trabecular meshwork cells [50], (3) retinal pigment epithelial cells [52], and (4) cornea sclera margin, ciliary margin, and retina following transplantation of hematopoietic stem cells [44]. All images are reproduced with permission under the Creative Commons Attribution 4.0 International license (http://creativecommons.org/licenses/by/4.0/) or from the indicated citations ([40] AAI is the copyright holder and [44] ARVO is the copyright holder).

The current knowledge of TNTs in specific ocular tissues is summarized in Figure 6. While the discovery of TNTs has challenged existing dogma and opened up new avenues for research into how cells communicate, there are still many outstanding questions that need to be addressed. Studies on signals, mechanisms, and/or microenvironments that initiate their creation, as well as determining the molecules involved in their elongation, stability, and collapse, will likely yield novel ways that we can control TNTs. Improvements in high-resolution, intravital imaging techniques and development of specific fluorescent reporter mice will enable further investigation into the physiology of TNT formation in ocular tissues *in vivo*. Targeted inhibition of key proteins, or RNA silencing of genes involved in TNT formation, in specific tissues and cell populations will also lead to further understanding of the function of TNTs during ocular homeostasis and during disease. This should open new avenues of research to design novel therapies for the treatment of a wide range of eye diseases.
